# Lacerated Cervix

**Published:** 1901-09

**Authors:** 


### Lacerated Cervix.

---

Deaver, of Philadelphia, in a recent article, claims that a lacerated cervix in women who have borne children should usually be considered more of a normal than a pathological condition. In the absence of special indications, such a cervix had better be let alone. If, however, a lacerated cervix be extensive enough to permit gaping of the edges and consequent exposure of the cervical mucous membrane to injury, or if ulceration be present, or if the scar tissue is hard and in excessive amount, or if any of these conditions give rise to subinvoluton or marked reflex symptoms, then operation is indicated. If, in addition to any of the above conditions, there is a history of hereditary tendency toward malignancy, there is the strongest indication for operation. A patient with a family history of carcinoma, presenting the above conditions, should be operated upon at the earliest possible moment; and this should be repeated after subsequent labors if the cervix be torn again, as it is likely to be. As strong as these indications are for operative interference, we are not justified in instituting them unless there is freedom from all pelvic inflammatory processes or their results, salpingitis, pyosalpinx or adhesions offer strong contra-indications. Under these circumstances, abdominal section for the correction of intra-abdominal trouble should follow immediately the repairing of the lacerated cervix. If the cicatricial tissue in a lacerated cervix involve the supra-vaginal cervix, it may be sometimes impossible to remove it entirely except by high amputation of the cervix, with freeing of the bladder and rectum; if, under these circumstances, there is a history of a hereditary tendency to malignancy, or if the patient be near or undergoing the menopause, vaginal hysterectomy may be considered the more rational procedure. He also thinks that great care must be exercised in endometritis to properly prepare the endometrium, if this be possible, prior to the narrowing of the cervical canal, so as to provide adequate drainage, or, in other words, to decrease the discharge so that the new and narrow canal will carry it off. Aseptic details in any one of these operations are as necessary as in any in the realm of surgery. He calls attention again to the fact that a latent salpingitis can be converted into an active one by the introduction of sepsis through instruments or intra-uterine douching, or the spread of sepsis from an infected uterine cavity, or the breaking up of peri-uterine adhesions, liberating septic foci which have been imprisoned. He further says that adhesions may be torn by bringing the uterus down to the vulvar orifice; therefore, the tenaculum should not be used to make traction during dilatation or repairing the cervix, but only to steady the uterus. He regards washing out the uterus, except in septic conditions, and also plugging the uterine cavity with iodoform gauze, as vicious practices too often capable of exciting salpingitis. If the discharge arising from an endometritis shows the presence of gonococci, curettement is positively contra-indicated, for it is certain that such a procedure will most probably light up an active gonorrhea, which will show marked tendencies to spread with consequent tubal involvement.—*Am. Surgery and Gynecology.*

